# DASP3: identification of protein sequences belonging to functionally relevant groups

**DOI:** 10.1186/s12859-016-1295-z

**Published:** 2016-11-11

**Authors:** Janelle B. Leuthaeuser, John H. Morris, Angela F. Harper, Thomas E. Ferrin, Patricia C. Babbitt, Jacquelyn S. Fetrow

**Affiliations:** 1 Molecular Genetics and Genomics Program, Wake Forest University, Winston-Salem, NC 27106 USA; 2 Department of Pharmaceutical Chemistry, University of California San Francisco, San Francisco, CA 94158 USA; 3 Department of Physics, Wake Forest University, Winston-Salem, NC 27106 USA; 4 Department of Bioengineering and Therapeutic Sciences, University of California San Francisco, San Francisco, CA 94158 USA; 5 Department of Chemistry, University of Richmond, Richmond, VA 23173 USA; 6 Present address: University of Richmond, Gottwald Hall C302, Richmond, VA 23173 USA

**Keywords:** Active site profiling, Protein function annotation, Functionally relevant clustering, Misannotation

## Abstract

**Background:**

Development of automatable processes for clustering proteins into functionally relevant groups is a critical hurdle as an increasing number of sequences are deposited into databases. Experimental function determination is exceptionally time-consuming and can’t keep pace with the identification of protein sequences. A tool, DASP (Deacon Active Site Profiler), was previously developed to identify protein sequences with active site similarity to a query set. Development of two iterative, automatable methods for clustering proteins into functionally relevant groups exposed algorithmic limitations to DASP.

**Results:**

The accuracy and efficiency of DASP was significantly improved through six algorithmic enhancements implemented in two stages: DASP2 and DASP3. Validation demonstrated DASP3 provides greater score separation between true positives and false positives than earlier versions. In addition, DASP3 shows similar performance to previous versions in clustering protein structures into isofunctional groups (validated against manual curation), but DASP3 gathers and clusters protein sequences into isofunctional groups more efficiently than DASP and DASP2.

**Conclusions:**

DASP algorithmic enhancements resulted in improved efficiency and accuracy of identifying proteins that contain active site features similar to those of the query set. These enhancements provide incremental improvement in structure database searches and initial sequence database searches; however, the enhancements show significant improvement in iterative sequence searches, suggesting DASP3 is an appropriate tool for the iterative processes required for clustering proteins into isofunctional groups.

**Electronic supplementary material:**

The online version of this article (doi:10.1186/s12859-016-1295-z) contains supplementary material, which is available to authorized users.

## Background

As protein sequence databases have exponentially increased in size [1], the demand for automated methods to accurately characterize protein function has soared. Many automated methods utilize full sequence similarity or full structural similarity to classify proteins based on function; however, studies have shown major databases are plagued by misannotation [2–5], often due to annotation transfer between proteins based on sequence similarity [5, 6]. Ideally, automated methods can be developed to identify detailed protein function such as that annotated in the Structure-Function Linkage Database (SFLD), a high quality dataset developed using a thoughtful combination of computation and expert curation [7]. One strategy for function prediction involves subdividing large protein sets into small groups based on functional similarity. In this way, protein function can be transferred from one protein in the group to all other proteins in the group if the groups accurately portray functional relationships. Notably, it is crucial to fully understand the level of molecular functional similarity within the groups before engaging in detailed molecular function annotation transfer to avoid further misannotation.

Many methods that group proteins by sequence or structural similarity are useful for inferring broad functional similarities to the degree that such similarities track with functional similarities. Resources such as PFAM [8], which identifies sequence similarities using multiple sequence alignments and hidden Markov models, and CATH [9] and SCOP [10], which classify proteins based on domain structural similarities, offer examples. Because sequence and structure similarities do not always track with molecular function, these classification systems do not subdivide protein sets to a detailed level of protein function. The functional groups identified in these methods most closely resemble the broadest level of functional detail in the SFLD – the protein superfamily.

More recently, methods such as GeMMA [11] and SCI-PHY [12] were developed to group proteins by functional relationships; yet, early results suggested that these methods distinguish more functional classes within protein superfamilies beyond what current evidence can support [11]. For example, the enolase superfamily was used as a test set for both GeMMA and SCI-PHY and has been comprehensively studied by the SFLD curators. GeMMA identifies 143 functional groups (using superfamily-specific parameters) and SCI-PHY identifies 201 functional groups for the enolase superfamily; conversely the SFLD contains just 28 functional families, each family representing a distinct chemical mechanism as evaluated by expert curators and, in some cases, experiment [13–17]. Developing automated methods to accurately group proteins by detailed function remains a significant hurdle-the level at which clustering is evaluated is currently an area of study.

To overcome the limitations of full sequence similarity methods in tracking detailed molecular function, active site profiling was developed to compare proteins based on structural features at their functional sites [18]. This method was compared to the clustering of proteins using PRINTS [19, 20], BLOCKS [21], BLAST [22, 23], and PFAM [8] and was further tested on the difficult assignment of classifying kinases by function [18]. A software tool, Deacon Active Site Profiler (DASP), was developed to implement active site profiling so that it allowed searching PDB and GenBank databases for proteins with sequences containing active site features similar to the features of the query set [24]. DASP was evaluated in early studies on the cyclooxygenase family of proteins [25] and, more extensively on the peroxiredoxin (Prx) superfamily, demonstrating its ability to identify proteins belonging to distinct functional groups in comparison to an expertly-defined input set [26]. A direct comparison with PSI-BLAST on a set of 58 proteins whose functional family had been defined by experimental biochemical analysis demonstrated the superior performance of DASP at identifying detailed functional relationships between proteins without identifying false positives at significant scores [26]. Most recently, the approach was used to identify targets sharing specific active site features within the cytochrome P450 protein superfamily, with subsequent experimental verification of results [27].

For enzymes, sequence similarity networks have also recently been used to subdivide large protein superfamilies [28, 29] into subgroups useful for generating hypotheses about the degree of sequence similarity that correlates with detailed differences in molecular function. Later work incorporating similarities inferred from active site motifs identified from active site profiling showed improvement in capturing more detailed functional relationships [30]. To analyze the differences between full sequence, full structure, and active site similarity, in that work, networks were created for proteins of known structure for the well-studied SFLD superfamilies such as the enolases. While full sequence- and full structure-based networks corresponded well with known functional relationships at a broad level of functional detail (superfamily and often subgroup), the relationship was often less precise at the most detailed level of function (SFLD family). While active site similarity-based networks, which identify similarity based on active site features, tracked more closely with known functional annotations for experimentally characterized proteins at the most detailed level of function (reaction specificity), identifying a single threshold to define functional relationships was still difficult [30]. Ultimately, accurate identification of proteins that share mechanistic features and, thus, chemical mechanism, remains a challenge.

The combination of active site profiling and protein clustering has shown notable promise in identifying detailed protein functional relationships from a single search of the sequence database [26, 27]. However, DASP searches are based on single searches of the sequence database using profiles built for proteins of known structure, a very limited input set. To accomplish the broader goal of clustering the universe of proteins in ways that track with detailed molecular function, we developed an iterative search process. At each search iteration, the process adds members to clusters and evaluates whether each cluster contains more than one functionally relevant group. We have developed two such automatable processes: one to cluster protein structures and another to cluster protein sequences. Both methods have demonstrated promising ability to identify functionally relevant groups in the well-studied enolase and Prx superfamilies (manuscripts under review).

Both of these processes use iterative DASP searches of databases with each iteration building on the results of the previous search. This iterative searching illuminated limitations of the original DASP algorithm that only became apparent through search iterations. In this contribution, we describe the enhancements to the DASP algorithm to overcome these limitations and we demonstrate how the software modifications changed DASP scores and output results. The enhancements were built in two steps: three incremental enhancements to produce DASP2 and three more significant enhancements to produce DASP3. Validation of the significant algorithmic changes implemented into DASP3 is presented here, and their application to the iterative search processes demonstrates the improved quality of searches. (For completeness, the incremental software modifications to produce DASP2 and results showing enhanced algorithm speed are documented in Additional file 1).

## Implementation

### DASP algorithm overview

**Fig. 1:**
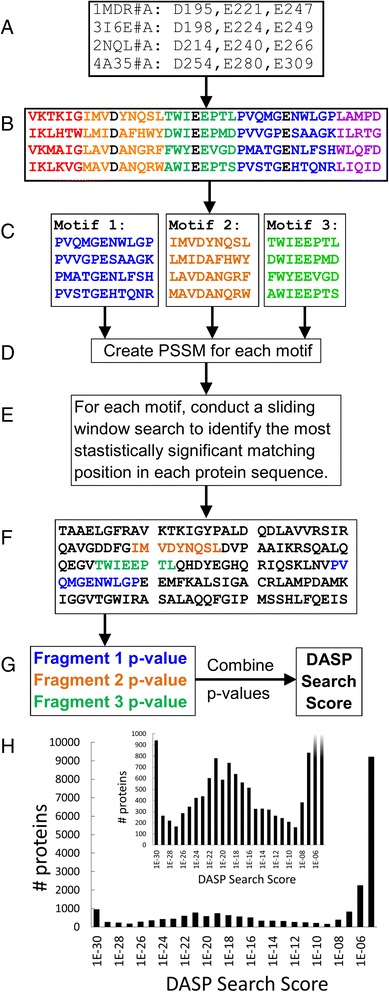
DASP flowchart for identifying active site profiles and searching GenBank. Key catalytic residues are chosen for each protein (**a**) and used to identify sequence fragments in the active site profile (**b**). The fragments are separated into motifs (**c**) which are used in the GenBank search. A PSSM is calculated for each motif (**d**) and a sliding window search (**e**) is utilized to identify the best positional match for each motif in each protein sequence by calculating a *p*-value at each position (**f**). The *p*-values for each matched motif are combined to calculate a final DASP search score (**g**). The distribution of DASP search scores for a given GenBank search are visualized using a histogram with DASP search score on the x-axis and the number of proteins identified on the y-axis (**h**). The inset shows the same histogram with the y-axis capped at 1000 proteins to better highlight the distribution of DASP search scores

As previously published [25, 26], the DASP input is a list of proteins with corresponding functionally relevant key residues (typically three per protein) (Fig. 1a); these residues define the active site microenvironment. For each protein, all residues with their center of geometry within 10 Å of the center of geometry of any key residue are extracted and concatenated N- to C-terminus to create the active site *signature*. The active site signature identifies the features that define a given protein functional site. Fragments containing one of the key residues are defined as key-residue fragments. The signatures from all input proteins are aligned to create an *active site profile* (ASP, Fig. 1b). The ASP allows identification of commonalities and differences at the active site in the set of proteins. Motifs are identified from aligned fragments (ignoring fragments of length one and two as they are too short to form meaningful motifs) and arranged by length (Fig. 1c). A position specific scoring matrix (PSSM) [31] is calculated for each aligned motif (Fig. 1d), which captures the conserved and less conserved features at each position in the active site across the protein set contained in the profile.

To search the PDB and GenBank databases for proteins with similar active site features, each motif in the ASP, beginning with the longest motif, is aligned to each continuous fragment in a given search sequence using a sliding window method, as previously reported [25]. A *p*-value is calculated for every possible match position (Fig. 1e); this *p*-value takes into account the similarity between the sequence fragment and the motif PSSM, given the motif length, the protein length, and the background frequency of each residue in GenBank. The *p*-value represents statistically the quality of the match between the query motif and the sequence fragment compared to the match of the query motif to a random sequence fragment. The match position in the search sequence corresponding to the most significant *p*-value is identified for this first, longest motif and the process is repeated with the next longest PSSM motif (Fig. 1f), with the stipulation that no two motifs can be matched at overlapping positions. Once all motifs in the profile have been aligned to the protein sequence, the *p*-values for each motif are combined using QFAST [32] to produce a DASP search score, a combined *p*-value which represents the statistical probability of the protein sequence matching all input motifs, given the null hypothesis of the input motifs matching a random sequence (Fig. 1g). This process is repeated for every protein sequence in the database; more details of the scoring calculations can be found in Additional file 1. The search output is visualized as a histogram showing the distribution of DASP search scores for every protein in the database (Fig. 1h; only DASP scores ≤1E-5 shown).

### DASP3 Implementation

During development of iterative search processes that cluster proteins into isofunctional groups, three limitations of the DASP algorithm were identified. Three enhancements were explored to solve these iterative search limitations: minimum useable motif length, motif alignment process within the profile, and motif length used in the search.

**Fig. 2:**
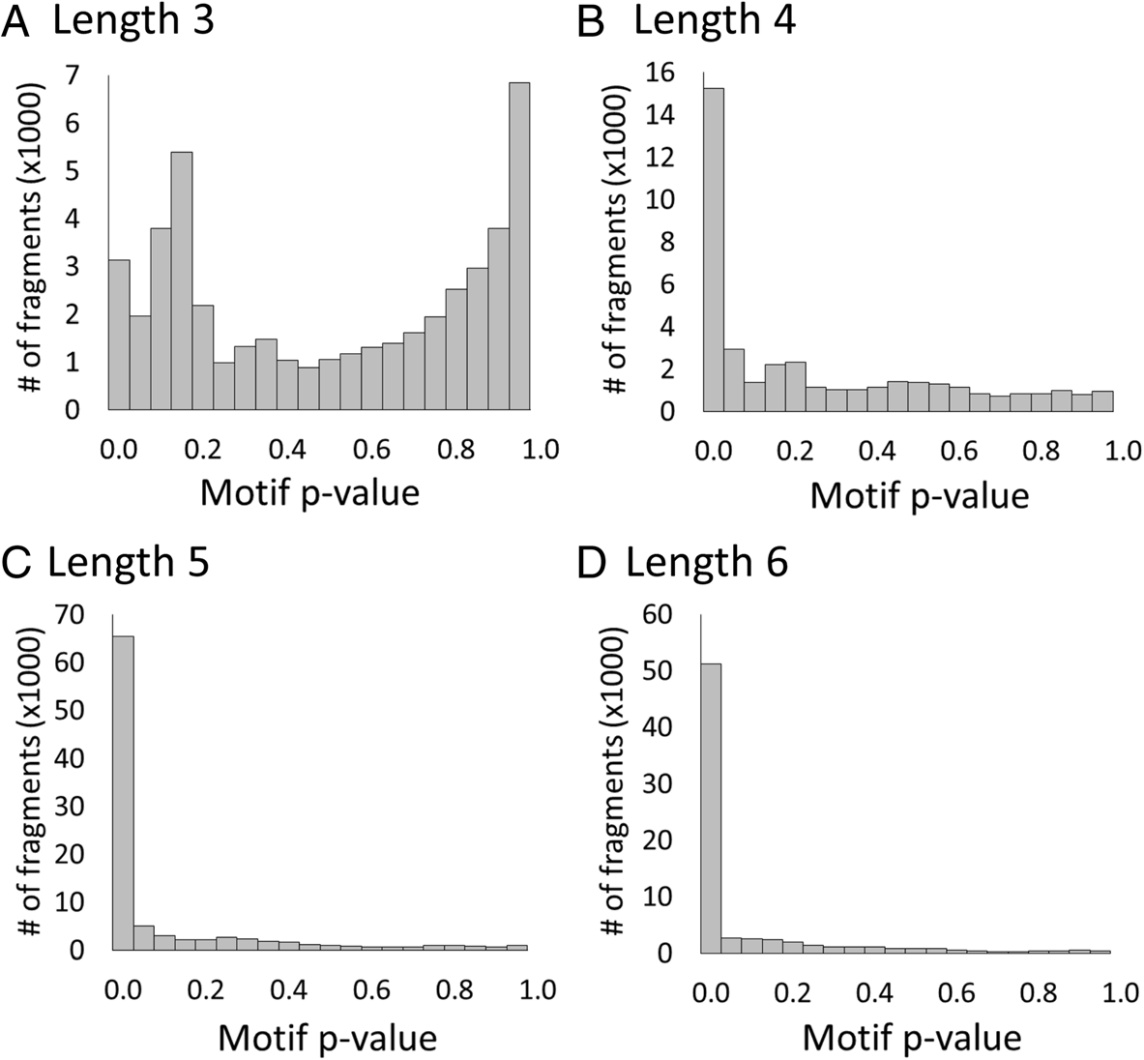
Length three fragments produce less significant *p*-values than fragments of length four through six. The motif *p*-values for fragments of length three residues (**a**), four residues (**b**), five residues (**c**), and six residues (**d**) are shown as histograms with motif *p*-values on the x-axis and the number of fragments on the y-axis. This data comes from a test set of 74 GenBank searches from six diverse SFLD protein superfamilies

The impact of three-residue fragments on search accuracy was evaluated first. It was previously demonstrated that DASP search scores accurately distinguish true positives from false positives [26]; however, a significant overall score does not guarantee significant *p*-values for individual fragments. Detailed fragment analysis revealed three-residue fragments were not always identified in the proper position during the GenBank searches. To investigate the relevance of three-residue fragments, 74 GenBank searches were performed using functionally relevant groups from six gold-standard superfamilies (crotonase, enolase, glutathione transferase (GST), peroxiredoxin, radical SAM, and vicinal oxygen chelate (VOC)) in the SFLD [7]. Over 92,000 proteins were identified at DASP search scores ≤1E-8. Analysis of the individual fragment *p*-values indicates the *p*-value distribution for three residue fragments is bimodal while the distributions of *p*-values for four-, five-, and six-residue fragments are positively skewed (Fig. 2).

**Fig. 3:**
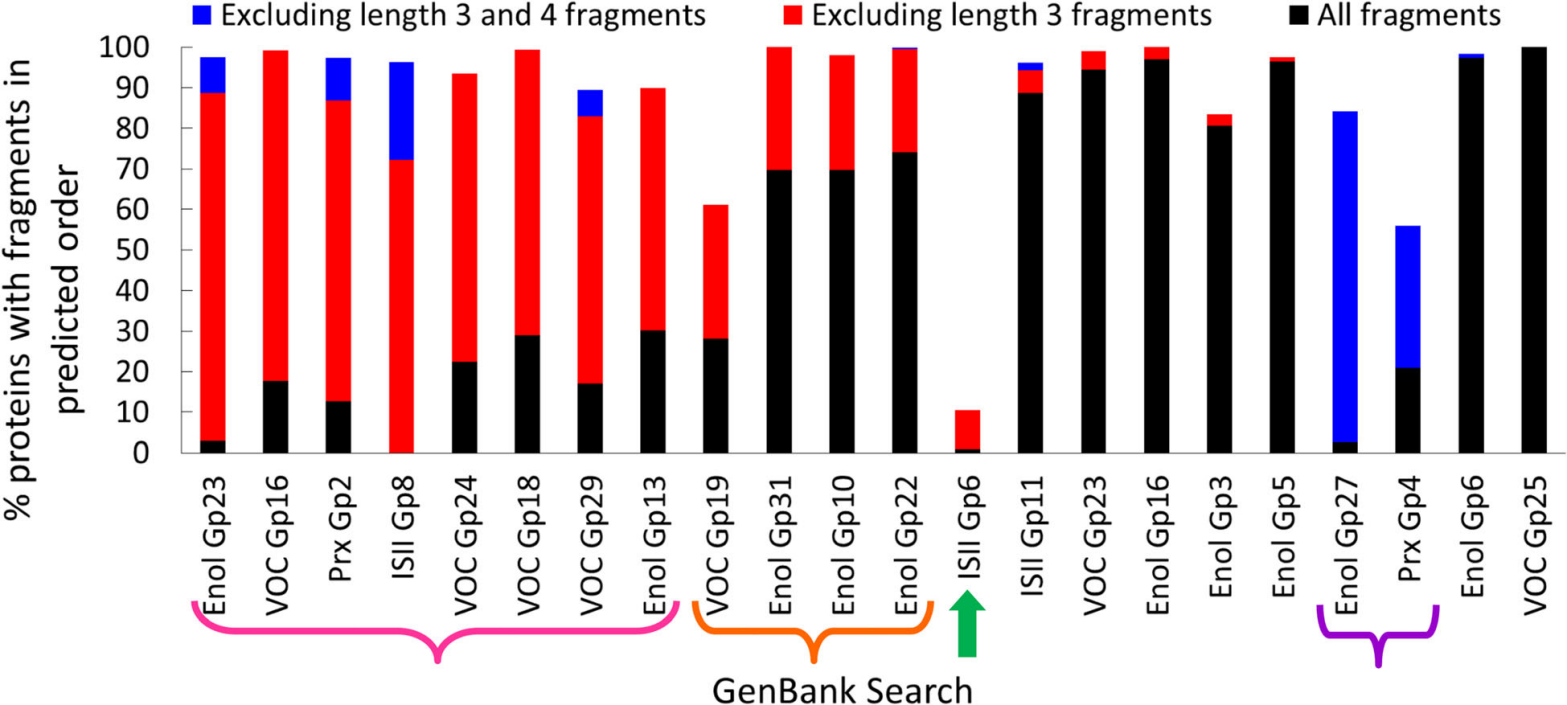
Three residue fragments are responsible for the majority of incorrect fragment order. The percent of proteins identified in GenBank searches with all fragments in the predicted N- to C- terminal order is plotted for 22 GenBank searches from four diverse SFLD protein superfamilies. The percent where all fragments are in the predicted order is shown in *black* bars, whereas *red* bars show the additional percent achieved when fragments of length three are not considered in the analysis and *blue* bars show the additional percent when both fragments of length three and four are not considered in the analysis. The *green* arrow and colored braces are discussed in the text

We also observed that three-residue fragments identified at less-significant scores (Fig. 2a, right) are often identified at an incorrect position in the sequence. To investigate how often this occurred, the N- to C- terminus order of identified fragments in each GenBank search was compared to the N- to C- terminus order of the input ASP motifs. Assuming no major structural rearrangements in these superfamilies, fragments identified in a different N- to C- terminus order than the ASP motifs suggests incorrectly identified fragments. Therefore, the percent of proteins with fragments in the predicted order was calculated for 22 GenBank searches across four superfamilies in three ways: using all fragments, removing three-residue fragments, and removing both three- and four-residue fragments (Fig. 3). When three-residue fragments are excluded from analysis, eight searches demonstrate ≥50 % increase in identifying fragments in the predicted order (Fig. 3, pink bracket). Additionally, four other searches demonstrate between 25 and 50 % increase in identifying fragments in the correct order (Fig. 3, orange bracket). Together, these results suggest that these short three-residue fragments are often being identified improperly in GenBank searches. Conversely, fragment identification is improved by ≥25 % in just two groups when length four fragments are removed (Fig. 3, purple bracket). In both of these groups, the four-residue motif is poorly formed and contains many gaps resulting in inaccurate fragment identification, which is addressed by the third modification discussed below.

The lone outlier in these data is ISII Gp 6, in which just 10 % of proteins are identified with fragments in the correct order (Fig. 3, green arrow). In this group, the ASP is misaligned due to structural variation among the input structures which causes two motifs to overlap in the majority of proteins. Because DASP-identified fragments cannot overlap, the shorter of the two fragments is identified incorrectly 87 % of the time. (Note: this problem has been solved in DASP3. Fragment overlap is identified at the beginning, during ASP creation, and an error is returned; the user must identify different key residues prior to completing the search.)

Most groups benefit significantly from the removal of three-residue fragments in the searches, but removing four-residue fragments does not considerably improve the searches. Furthermore, results from these searches show that significant important functional information can be lost when four-residue fragments are removed, such as the GGLG motif in the Prx superfamily [33]. Thus, DASP3 excludes fragments of three residues (or fewer) in ASP creation and motif identification.

The second DASP3 modification focused on creating ASP alignments that more accurately reflect the active site similarity between proteins. In DASP, the ASP is an alignment of complete active site signatures (concatenated fragments) which, because of variable fragment numbers and lengths, results in misaligned motifs. In DASP3, the active site fragments are aligned individually, rather than as complete signatures. Fragments containing key residues are aligned with analogous fragments first, then all other fragments are placed in N- to C- terminus order. However, if the number of fragments containing key residues is inconsistent across the group of proteins, the program terminates and reports an error.

**Fig. 4:**
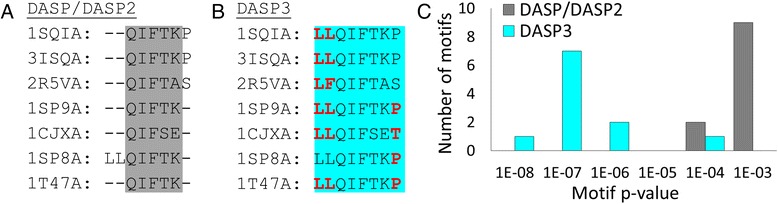
Motif *p*-values are more significant when ASP motifs are lengthened prior to database searching. In DASP/DASP2, motifs are identified from the shortest fragment in the set (**a**) while the longest fragment in the set determines motif length in DASP3 (**b**). Colored areas indicate the motif used in the database search. The motif *p*-values from the GenBank searches are visualized with a histogram (**c**) where grey indicates the DASP/DASP2 values and cyan indicates the DASP3 values. Each motif *p*-value corresponds to the significance with which it was identified in one protein (11 proteins were identified in the search)

The final enhancement targeted the length of motifs determined from the ASP. In the original implementation, motifs were extracted and compared and the shortest fragment from each aligned set defined the length of that motif (Fig. 4a). This implementation discarded potentially important active site detail due to slight variation between protein structures. During iterative searches, we discovered that this can cause some motifs to decrease in length. To preserve useful active site information, DASP3 defines motif length by the longest fragment in the set. Each shorter fragment is extended (using information from the complete protein sequence) to the length of the longest fragment (Fig. 4b). Ultimately, the longer motifs, which contain more active site sequence information, produce more significant *p*-values when used in GenBank searches (Fig. 4c). In addition, fragment length does not degrade during iterative searches.

Validation was performed to compare DASP3 with previous versions of the software and to analyze how well DASP3 identifies all members of known functionally relevant groups. First, a paired analysis was completed to compare DASP3 scores with DASP/DASP2 scores. As DASP and DASP2 search scores are essentially identical (see Additional file 1 for details), a single comparison was necessary to demonstrate the change in DASP3 search scores from previous versions. Subsequently, the ability of DASP3 to identify known functionally relevant groups of protein structures using an iterative clustering method was assessed. Finally, we analyzed the performance of DASP3 compared to previous versions in identifying functionally similar protein sequences using both a single GenBank search and an iterative sequence search process.

## Results

### DASP3 search scores segregate true positives from false positives to a greater extent than previous versions

Because DASP search scores are a critical part of our methods for clustering proteins into functionally relevant groups, it is paramount to understand how DASP3 search scores compare to DASP/DASP2 search scores. ASPs of previously identified functionally relevant groups were used to search both the PDB and GenBank databases with DASP2 and DASP3 for proteins that share active site features. Functionally relevant groups are defined here as groups identified by our Two Level Iterative clustering Process (TuLIP), which are largely equivalent to the subgroups and families annotated by SFLD curators (manuscript under review). Ideally, when each protein group is used to search the PDB, every protein in the group should be identified with a DASP search score more significant than the trusted cutoff and no other proteins should be identified with significant scores. The trusted cutoff for TuLIP is ≤1E-10 for most of the groups but can sometimes vary between ≤1E-8 and ≤1E-12.

**Fig. 5:**
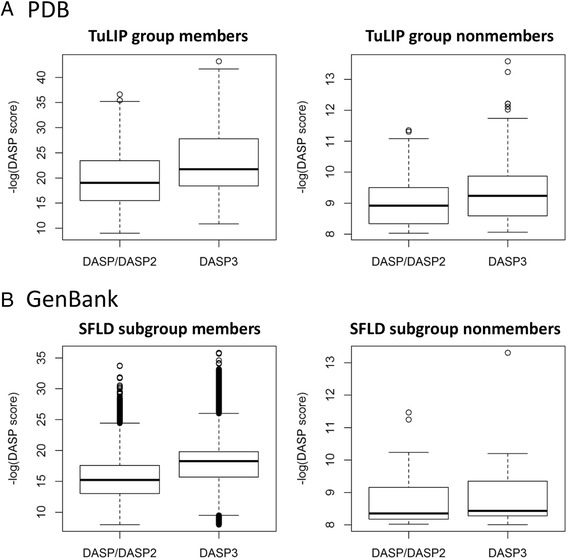
DASP3 PDB and GenBank true positive search scores are significantly improved compared to DASP/DASP2 scores. PDB (**a**) and GenBank (**b**) DASP search scores are visualized as boxplots for true positives (left) and true negatives (right). For the PDB searches, true positives are proteins which are a part of the TuLIP group used as input to the search (members) while true negatives are proteins which are not a part of the TuLIP group used as input to the search (non-members). For the GenBank searches, true positives are proteins annotated to the subgroup in which the input proteins are annotated (members) while true negatives are proteins annotated to a different subgroup than that which the input proteins are annotated (non-members); annotations were identified from the SFLD

Active site profiles from 79 functionally relevant groups identified from five SFLD superfamilies (crotonase, enolase, GST, radical SAM, and VOC) and one expertly curated superfamily (peroxiredoxin) were used to search the PDB database. Each search was performed using both DASP2 and DASP3. The search results demonstrate DASP/DASP2 and DASP3 identify all group members at search scores ≤1E-8, but the DASP3 search scores are more significant by 2.97 orders of magnitude, on average (Fig. 5a, left). Paired *t*-test calculations indicate group member (true positive) DASP search scores are significantly improved between DASP/DASP2 and DASP3 for each superfamily with all *p*-values ≤1E-4 (Additional file 1: Table S1); DASP search scores for group non-members are not significantly changed between DASP2 and DASP3 (Fig. 5a, right).

**Fig. 6:**
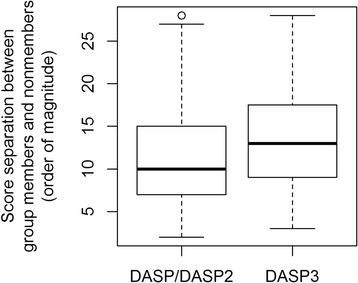
DASP3 separates true positive and false positive search scores more than previous versions. The magnitude difference between the least significantly scoring true positive (TuLIP member) and the most significantly scoring true negative (TuLIP non-member) is shown as a boxplot for 79 PDB searches completed with both DASP/DASP2 and DASP3. The PDB searches only reported proteins identified with scores ≤1E-5, so in cases where no TuLIP non-members were identified with such scores, the best scoring TuLIP non-member was assumed to have a DASP search score of 1.01E-5 for calculation purposes

Notably, as in previous versions, the group members and non-members are separated by at least two orders of magnitude in all 79 DASP3 searches (Fig. 6), demonstrating DASP3 can distinguish self and non-self across the isofunctional groups in the six diverse superfamilies. Furthermore, the average separation between the least significantly scoring group member and most significantly scoring non-member increases from 11 orders of magnitude in DASP/DASP2 to 13 orders of magnitude in DASP3 (Fig. 6), suggesting DASP3 separates true positives and false positives better than early versions of the software. The line of separation between group members and non-members falls in the range 1E-8 to 1E-12 for all 79 groups in DASP/DASP2; similarly, in DASP3, the line of separation is between 1E-10 and 1E-14 for all groups, as expected from the search score significance shift. The DASP search score which separates group members from group non-members is remarkably consistent, corroborating previous data suggesting significance thresholds for DASP search scores are less dependent on superfamily than other common classification methods [26].

To validate DASP3 performance in GenBank searches, 12 ASPs (from the enolase, ISII, and Prx superfamilies) corresponding to SFLD-defined functionally relevant groups were used to search GenBank with both DASP2 and DASP3 (Additional file 1: Table S1). Proteins were deemed true positives or false positives based on membership in the SFLD functional group represented by the input set, as it has been previously shown that proteins identified at significant DASP search scores are almost always annotated to the SFLD functional group of the input set [26]. Any proteins identified in GenBank searches which are not annotated in the SFLD were not used in this analysis as accurate functional group membership cannot be determined.

Similar to the PDB searches, DASP3 search scores for each subgroup are more significant by an average of 2.81 orders of magnitude compared to DASP/DASP2 (Fig. 5b, left); Wilcoxon rank test *p*-values are < 2E-16 for each superfamily, indicating significant improvement of DASP3 search scores (Additional file 1: Table S1).

Further, the false positive discovery rate [FP/(TP + FP)] for both DASP/DASP2 and DASP3 is < 0.5 % at a generous threshold ≤1E-8 and < 0.01 % at a trusted threshold ≤1E-12. In this analysis, false positives are defined as proteins that are members of SFLD functional groups not included in the input profile. While the false positive discovery rate is slightly higher for DASP3 (Fig. 5b, right), the difference is not statistically significant (*t*-test, *p* = 0.233). Taken together, these results demonstrate that DASP3 modifications enhance significance of the returned score and increase the score difference between true and false positives compared to previous versions of DASP.

### DASP3 accurately identifies known functionally relevant groups of protein structures using an iterative clustering process

The Two Level Iterative clustering Process (TuLIP), was recently developed to identify functionally relevant groups of protein structures using iterative clustering and DASP PDB searches (manuscript under review). In TuLIP, a protein cluster is defined as a functionally relevant group if the DASP PDB search returns only the proteins in the cluster at significant scores with no false positives. The process has demonstrated the ability to identify known isofunctional groups in multiple superfamilies (manuscript under review). However, major changes to the DASP algorithm could profoundly affect the groups identified in the TuLIP process. To analyze the impact of DASP modifications on TuLIP clustering, TuLIP was performed using both DASP2 and DASP3 on four superfamilies.

**Fig. 7:**
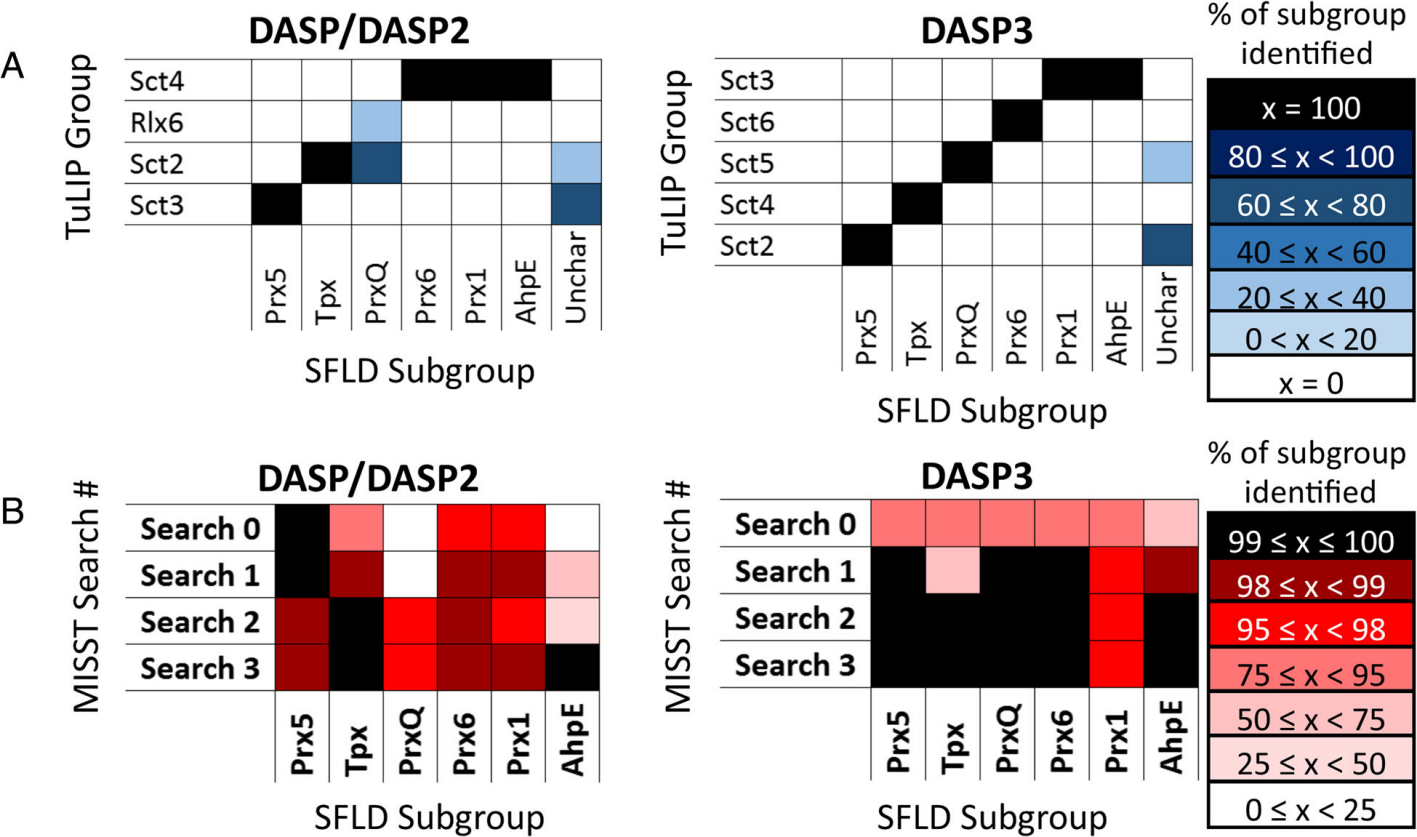
DASP3 identifies known Prx functional groups more accurately than previous DASP versions. **a** The clusters of the Prx superfamily identified by TuLIP are shown as heat maps for DASP/DASP2 and DASP3. The color of each box represents the percent of the known Prx isofunctional group identified by that TuLIP group, as shown by the legend. *White* boxes indicate no proteins from a functional group are in a given TuLIP group while *black* boxes indicate all proteins from a functional group are in a given TuLIP group. **b** Heat maps are used to demonstrate the coverage of each Prx functional group by DASP/DASP2 and DASP3 after each iteration of an iterative sequence search process. Box color indicates the percent of each Prx known functional group identified with DASP search scores ≤ 1E-8 at each iterative search level, as shown by the legend

Prior expert analysis separated the peroxiredoxin (Prx) superfamily into six subgroups [26]. DASP was previously able to identify these subgroups distinctly in both PDB and GenBank searches using a manually curated starting set [26]. When TuLIP was used with DASP/DASP2 to cluster the Prx proteins with no a priori knowledge (Fig. 7a, left), just one of the six subgroups was identified distinctly (Prx5 as Sct3). The Tpx subgroup was combined with some of the PrxQ proteins, while the remaining two PrxQ proteins formed another group. The final three subgroups (Prx6, Prx1, and AhpE) were combined into one TuLIP group (Sct4). Conversely, when DASP3 was used to perform TuLIP, four of the six subgroups (Prx5, Tpx, PrxQ, and Prx6) were grouped according to expert subgroup annotation, while the remaining two subgroups (Prx1 and AhpE) were combined (Fig. 7a, right). In this limited test case, the TuLIP-identified groups match the known functional groups more closely using DASP3 than early versions of the software.

While DASP3 improves Prx subgroup identification over previous versions, additional SFLD superfamilies (enolase, crotonase, and GST) showed minimal differences between the two versions. When DASP/DASP2 and DASP3 are used by TuLIP to cluster these three superfamilies into functionally relevant groups, 52 and 44 %, respectively, of TuLIP-identified groups correspond one-to-one with SFLD subgroups or families, a small difference that is not statistically significant (Additional file 1: Figure S2). The subgroups and families which are combined in DASP3, such as OSBS, dipeptide epimerases, and several in the glutathione transferase superfamily, are previously shown to be difficult to cluster [29, 34, 35].

Overall, the DASP/DASP2 and DASP3 results are consistent with regard to TuLIP-based functionally relevant clustering of the very limited proteins of known structure in the PDB. In some superfamilies, such as crotonase, enolase, and GST, DASP3 identifies functionally relevant groups in a similar fashion to early versions of the software. In other superfamilies, such as Prx, TuLIP is able to identify functionally relevant groups more accurately using DASP3.

### DASP3 accurately identifies known Prx isofunctional groups of protein sequences with one GenBank search

**Fig. 8:**
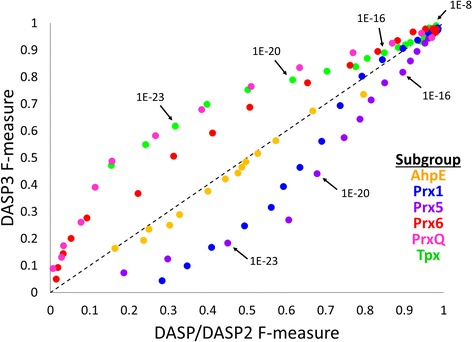
DASP3 identifies known Prx sequences in 6 isofunctional groups as well as previous software versions. The F-measure (harmonic mean of precision and recall) of DASP/DASP2 (x-axis) and DASP3 (y-axis) Prx searches is shown as a scatterplot for DASP search scores 1E-8 to 1E-25. The F-measure decreases with increasing DASP search score significance for all six groups, such that 1E-8 is in the upper right and 1E-25 is in the lower left. Color indicates known Prx subgroup, as shown by the legend

To analyze if DASP3 can identify all Prx protein sequences from a small set of known protein structures, the structures in the Prx superfamily were separated into six expertly-identified functionally relevant groups, as previously described [26]. Each of these six groups was used to search GenBank using DASP2 and DASP3. The F-measure was calculated at each DASP search score from 1E-8 to 1E-25 for both methods (Fig. 8). F-measure is the harmonic mean of precision [TP/(TP + FP)] and recall [TP/(TP + FN]; true positives, false positive, true negatives, and false negatives were defined by inclusion in the previously expertly-identified groups [26], as explained in detail by Knutson et al. (manuscript under review). All proteins identified in these GenBank searches that were not previously identified by Nelson et al. were not included in the F-measure calculations as group membership cannot be validated. F-measure scores range from 0 to 1 where 1 indicates the search identified all true positive proteins without identifying any false positive proteins at the given DASP search score threshold.

On average, the F-measure does not significantly differ between the DASP/DASP2 and DASP3 searches (Fig. 8). However, group-by-group analysis highlights some interesting behavior. In the AhpE subgroup, the F-measure does not significantly differ at any DASP search score threshold (Fig. 8, orange). For the Prx5 subgroup, the F-measure is consistently higher in DASP/DASP2 than DASP3, though the differences are small until more significant DASP search score thresholds (Fig. 8, purple). Similarly, the Prx1 subgroup results demonstrate a higher F-measure in DASP/DASP2 than DASP3 at DASP search scores ≤ 1E-17, but similar F-measure values at less significant thresholds. For both the Prx1 and Prx5 subgroups, the lower F-measure in DASP3 is due to the emergence of false negatives at more significant DASP search scores; that is, some proteins are identified at less significant DASP search scores in DASP3 than DASP2. Interestingly, the opposite pattern in F-measure values is observed for the Tpx, PrxQ, and Prx6 subgroups (Fig. 8, green, pink, and red, respectively). In these subgroups, the F-measure is higher in DASP3 than DASP/DASP2, particularly at more significant DASP search scores. Again, the presence of false negatives in DASP/DASP2 causes the lower F-measure scores as proteins are identified at less significant scores in DASP/DASP2 searches than DASP3 searches. The enhancements made to create DASP3 result in variable F-measure improvements on a group-by-group basis, but overall no significant differences are observed after a single GenBank search (paired *t*-test at significance thresholds ≤1E-14 for DASP/DASP2 and ≤1E-16 for DASP3; *p*-value = 0.12). Notably, DASP3 identifies a large proportion of known Prx sequences in the appropriate groups; the average weighted F-measure at significance thresholds of ≤1E-8 and ≤1E-16 is 0.97 and 0.72, respectively.

### DASP3 accurately and efficiently identifies known functionally relevant groups of protein sequences using an iterative sequence search process

As only structurally characterized proteins are clustered by TuLIP, GenBank searches are necessary to identify protein sequences belonging to each TuLIP group. Therefore, the Multi-level Iterative Sequence Searching Technique (MISST) was developed to iteratively identify protein sequences with active site similarity to a given functionally relevant group and, further, to determine when such groups should be subdivided based on active site similarity (manuscript under review). MISST has demonstrated the ability to identify, cluster, and subdivide the Prx superfamily and other superfamilies using DASP2. Since MISST is a key method in our software arsenal and relies on iterative searching of the sequence database, it was relevant to compare the results of the MISST process using DASP/DASP2 and DASP3. Consequently, MISST was applied to the functionally relevant groups in the Prx superfamily.

Three iterations of MISST were performed starting with the TuLIP groups identified by both DASP/DASP2 and DASP3 (Fig. 7a), as MISST is specifically designed to use TuLIP results as input. On the whole, both DASP/DASP2- and DASP3-identified MISST groups compare well with known functional groups (Additional file 1: Figure S3). After the first GenBank search (Search0), some subgroups are identified more completely by DASP/DASP2 (Prx1, Prx5, and Prx6), while some subgroups are identified more completely by DASP3 (Tpx, PrxQ, AhpE); this result supports the single-GenBank search result previously described: DASP3 does not significantly improve search results across the board after a single GenBank search. However, a greater percentage of each subgroup was identified (at DASP search scores ≤1E-8) in fewer DASP3 iterations compared to earlier versions (Fig. 7b). Notably, the PrxQ subgroup, which was difficult to identify using DASP/DASP2, was identified in full after just two iterative searches using DASP3.

Using more stringent thresholds to reduce the presence of false positives (≤1E-14 in DASP2 and ≤1E-16 in DASP3; see Fig. 5), we identified 21,632 total sequences with four iterations of DASP/DASP2 searches and 23,300 total sequences with four iterations of DASP3 searches, compared to the 3,390 sequences previously identified with a single DASP search of GenBank using a stringent threshold of ≤1E-10 [26]. Much of this increase is likely due to the five additional years of sequence addition to the database. However, some are likely newly identified sequences, given the added benefit of the modified algorithms and iterative searches. Given these and previous results, we expect the false positive rate at these score thresholds to be less than 1 %, but detailed analysis of these sequences is beyond the scope of this manuscript.

Together, these results show that beginning with DASP3-identified TuLIP groups, iterative DASP3 GenBank searches identify the six known Prx isofunctional groups to a similar standard as expert identification. Additionally, superfamily coverage through iterative searches is obtained more quickly using DASP3 than previous versions of the software. Though the enhancements produce incremental improvement for TuLIP clustering and single GenBank searches, the improvements sum to significantly improve the efficiency of identifying and clustering across the iterative process, which is necessary for complete functionally relevant clustering of protein superfamilies.

## Discussion

A key parameter in every classification method is the score threshold used to distinguish group members from non-members. In many methods, such as BLAST and PSI-BLAST, the trusted threshold is search dependent, making it difficult to identify a threshold for any given search without prior knowledge of the proteins that should be identified in that search. Previous work with DASP demonstrated a universal generous cutoff of ≤1E-8 for GenBank searches. At this threshold, all true positives were identified and the false positive rate was below 1 % [26]. Similarly, 1E-8 represents the separation of two modes in the DASP search score distribution, separating the tail (significant scores) from the bulk of the GenBank database scores (Fig. 1h). In our subsequent work with multiple, diverse superfamilies, 1E-8 has been confirmed as the generous threshold in which proteins identified with scores ≤1E-8 almost always belong to the superfamily of interest, but may not belong to the subgroup or family used in the search. Notably, this generous cutoff has consistently returned proteins belonging to the superfamily of interest, suggesting a universal score threshold for the DASP approach.

In Nelson et al.’s previous Prx analyses, a trusted threshold, at which false positives drop to zero, was identified as ≤1E-10 [26]. Subsequently, further work on the enolase superfamily determined a trusted score threshold of ≤1E-12 for initial GenBank searches with the goal of reducing false positives (manuscript under review). Iterative GenBank searches with the Prx superfamily, however, indicate that the trusted threshold should be ≤1E-14 beyond the first search iteration; as groups become more exclusive, the trusted threshold must be more significant to prevent false positives. In this work, DASP3 scores are two to three orders of magnitude more significant than DASP/DASP2 scores, suggesting the trusted threshold should be shifted when using DASP3. Therefore, the trusted threshold for initial DASP3 GenBank searches is ≤1E-14 and the trusted threshold beyond the first iterative search is ≤1E-16. Thus far, the generous and trusted thresholds have remained stable across the six diverse superfamilies analyzed, however, this remains to be analyzed further.

## Conclusion

In this contribution, we present DASP3, software for identifying sequences from databases that share motifs similar to a query active site profile. DASP3 is a modification of previously published software, DASP [24–26]. Limitations identified in DASP were addressed through six enhancements producing DASP3. This work demonstrated DASP3 is significantly more efficient and versatile than DASP, a requirement for the iterative processes used to cluster proteins into functionally relevant groups. DASP3 produced better separation between true positives and false positives than earlier versions of the software and showed improved ability to accurately and efficiently cluster the Prx superfamily into functionally relevant groups using two recently developed iterative processes. As an automated algorithm, DASP3 identifies isofunctional groups better than previous versions of the software and rivals expert manual curation in the SFLD.

## Availability and requirements

Project name: DASP3

Project home page: https://github.com/RBVI/dasp3

Operating system(s): Platform independent, but paths are configured for Linux

Programming language: Java

Other requirements: Java 1.5 or higher

License: GPL 3.0

Any restrictions to use by non-academics: None

## Additional file

Additional file 1: This file contains additional DASP methods, including the development and validation of DASP2, and supplemental figures with additional validation of TuLIP and MISST. (DOCX 1543 kb)

## Abbreviations

ASP: Active site profiling
DASP: Deacon active site profiler
GST: Glutathione transferase
ISII: Isoprenoid synthase type II
MISST: Multi-level Iterative sequence searching technique
MSA: Multiple sequence alignment
Prx: Peroxiredoxin
PSSM: Position specific scoring matrix
SFLD: Structure-function linkage database
TuLIP: Two Level Iterative clustering Process
VOC: Vicinal oxygen chelate
