# Supplementary material for: DASP3: identification of protein sequences belonging to functionally relevant groups
Source: BMC Bioinformatics. 2016 Nov 11;17:458. doi: 10.1186/s12859-016-1295-z (PMC5106842; doi:10.1186/s12859-016-1295-z)
Supplement: Additional file 1: — This file contains additional DASP methods, including the development and validation of DASP2, and supplemental figures with additional validation of TuLIP and MISST. (DOCX 1543 kb) [file 12859_2016_1295_MOESM1_ESM.docx]

**Additional Methods – detailed DASP search methods**

Algorithmic details of the DASP search score process are outlined by Huff et al. [1,2] and Nelson et al. [3]. Briefly, during a search of the PDB or GenBank database using DASP, each motif is used to find the best matching fragment in each protein sequence in the database. From the input ASP, a position specific scoring matrix (PSSM) is calculated for each motif, defining the likelihood that each residue is found in each position of the motif compared to the background presence of each residue in the database. To identify the motif matches in each protein, a sliding window search is completed for each motif compared to each protein. Starting with the longest fragment, every possible position for that fragment in the protein is scored against the PSSM by adding the matrix values in the PSSM (one value per column). To calculate a *p*-value from this data, the probabilities of finding each matrix value in each column used for the fragment match are multiplied together because each column is considered independent (a residue in one position does not affect which residue is in the next position). Finally, the *p*-values are normalized by the lengths of both the fragment and the protein sequences using the following equation

$$p_{seq}=1- {(1-p)}^{m-n+1}$$

where m – n + 1 is the number of possible positions a fragment of length n can be matched to a protein sequence of length m. The *p*-values for all fragments identified in one protein sequence are then combined using QFAST [4], a method specifically designed to combine *p*-values from sequence motif searching.

**Additional Data – DASP2 Implementation and Validation**

**DASP2 Implementation**

Three simple enhancements were made to create DASP2, a more versatile, efficient version of DASP. First, the input format was expanded to allow active site signature lists and pre-aligned ASPs for searching the database. This change supports iterative searches by allowing the fragments identified in one search to be used as the input for the next search. Second, modified amino acids in the PDB (e.g. selenomethionine) were changed to their more common counterpart rather than being identified with an X. A dictionary was created of the known modified amino acids and their corresponding (normal) one letter code to ameliorate this problem. Although this affected a small number of sequences, this change was important for accurate scoring of certain protein superfamilies, including the Prxs. Lastly, the search algorithm was parallelized to increase the efficiency of searching GenBank. Using Java thread pools, the database is essentially split into chunks and processed simultaneously on a single multicore processor.

**DASP2 GenBank searches are significantly more efficient than DASP GenBank searches**

To validate DASP2, twenty functionally relevant protein groups from three superfamilies (Prx, ISII, and crotonase) in the SFLD [5] were used to search the GenBank database with both DASP and DASP2. Among the 40,000+ proteins identified at DASP search scores <1E-8, only 20 (0.05%) were identified at scores more than one order of magnitude different between DASP and DASP2; all of those sequences contained modified amino acids represented with Xs in DASP. Further, the average run time of a GenBank search was decreased from just over 19 hours in DASP to just under 4 hours in DASP2 (Figure S1). A paired t-test comparing the run times of DASP and DASP2 produced a significant *p*-value of 5.96E-15, and each of the superfamilies individually produced significant *p*-values <0.001. These results demonstrate that DASP2 is significantly more efficient than DASP at identifying proteins with active site similarity across groups within three diverse protein superfamilies.

**Additional Figures and Tables**

**
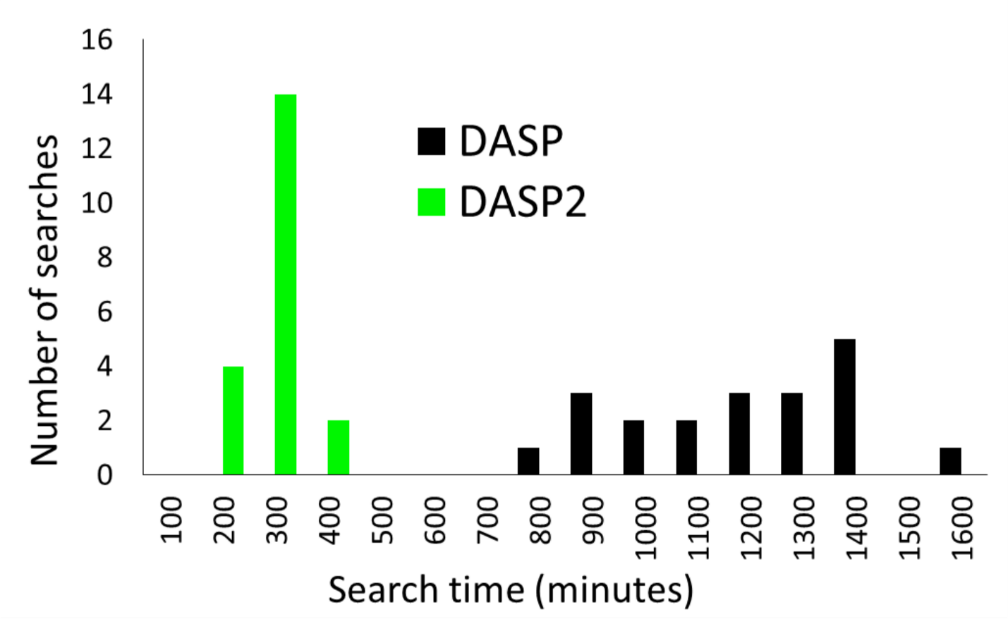
**

**Figure S1. DASP2 GenBank searches are significantly more efficient than DASP GenBank searches.** The GenBank search time (in minutes) for a test set of 20 GenBank searches is plotted as a histogram. The search times using DASP are shown with black bars and the search times using DASP2 are shown with green bars.

**Supplemental Table 1. DASP3 search scores are more significant than DASP/DASP2 search scores for all superfamilies.**

| **SFLD Superfamily** | **# groups used for PDB validation** | **PDB paired t-test p-value** | **# groups used for GenBank validation** | **GenBank Wilcoxon rank test p-value** |
| --- | --- | --- | --- | --- |
| Crotonase | 22 | 1.27E-11 | 0 | N/A |
| Enolase | 23 | 1.62E-10 | 6 | < 2.2E-16 |
| ISI | 13 | 1.68E-07 | 0 | N/A |
| ISII | 4 | 9.42E-05 | 4 | < 2.2E-16 |
| Prx | 4 | 9.24E-15 | 2 | < 2.2E-16 |
| VOC | 13 | 1.57E-11 | 0 | N/A |

Six superfamilies were used for PDB validation and three superfamilies were used for GenBank validation; the number of functionally relevant groups tested in each superfamily is shown. The DASP/DASP2 and DASP3 search scores for the PDB were compared using a one-tailed paired t-test. For GenBank, DASP/DASP2 and DASP3 search scores were compared using the Wilcoxon signed-rank test.


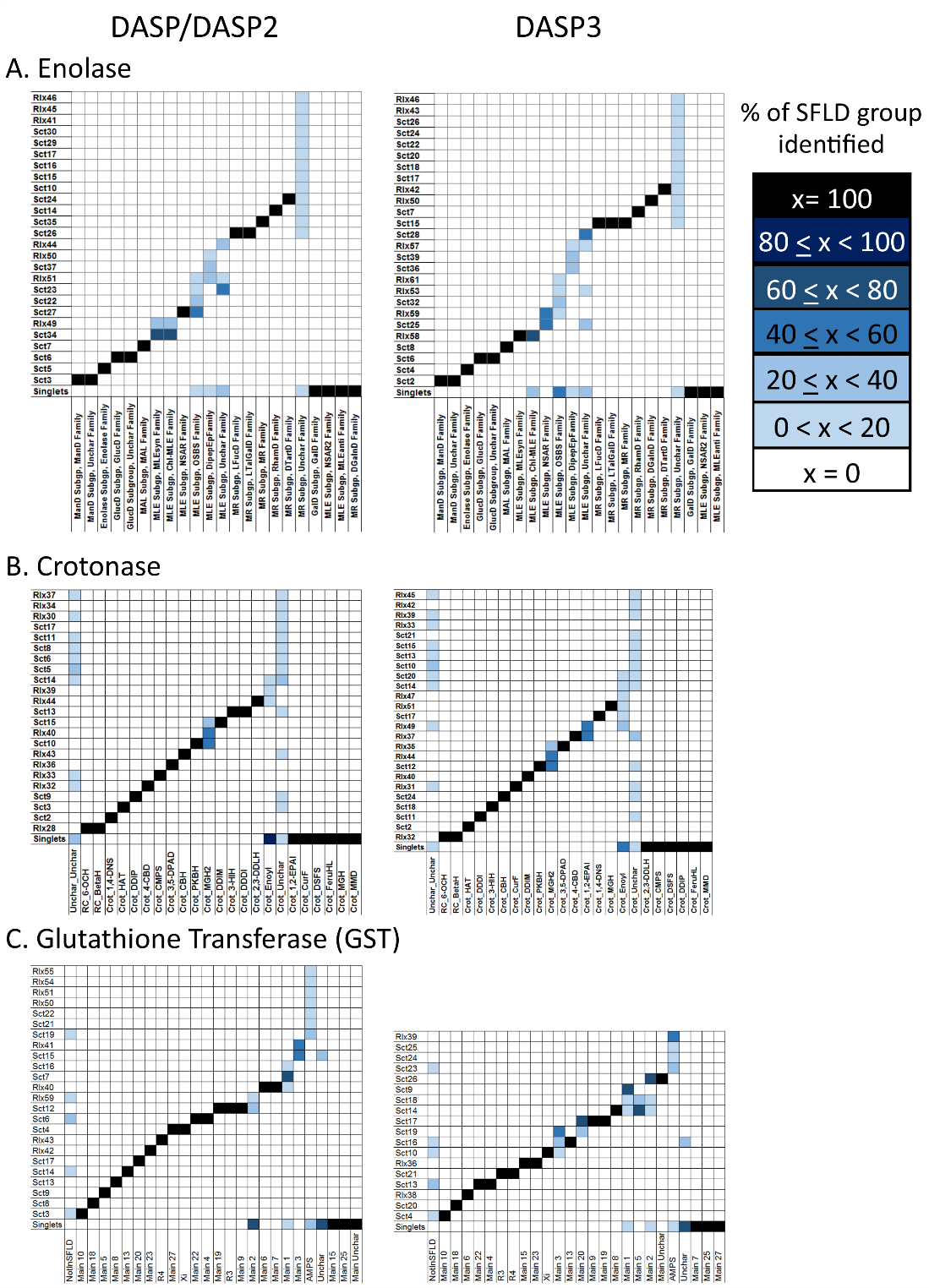


**Figure S2. DASP3 identifies known functionally relevant groups of protein structures as well as previous DASP versions.** Heat maps comparing TuLIP clusters to SFLD functional groups are shown for the enolase (A), crotonase (B), and glutathione transferase (C) superfamilies. For each heat map, the TuLIP clusters are represented by rows and the SFLD functional groups are represented by columns. Box color indicates the percent of each SFLD functional group found in each TuLIP cluster, according to the legend.


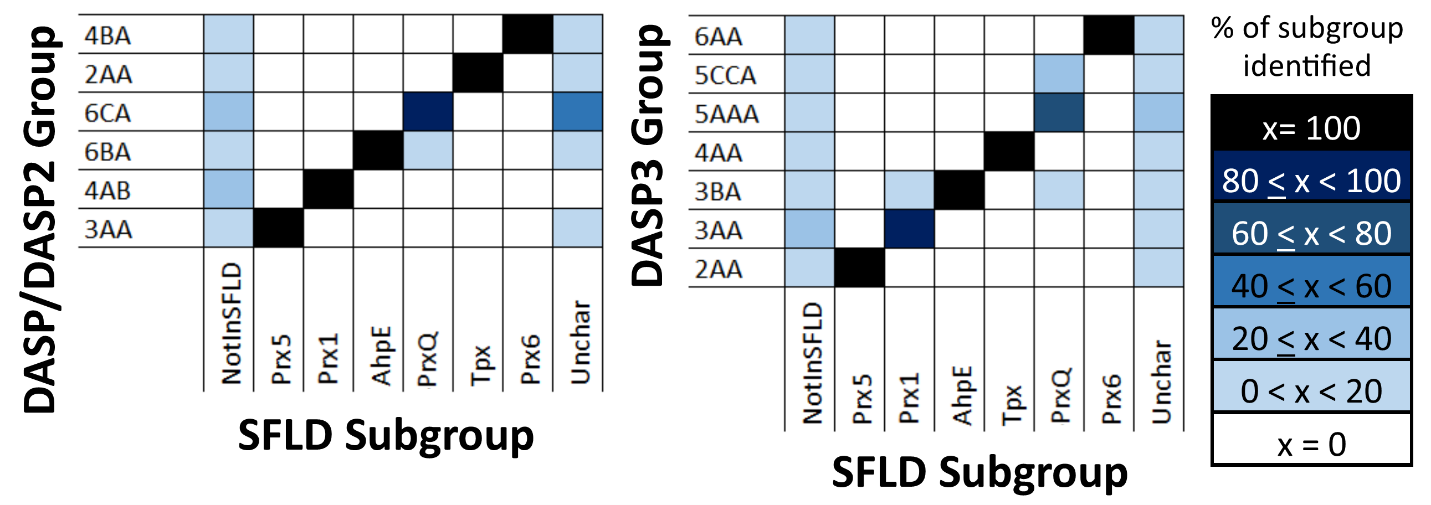


**Figure S3. DASP3 identifies known Prx isofunctional groups of protein sequences using an iterative sequence search process as well as previous DASP versions.** Heat maps demonstrate the correlation between DASP/DASP2 (left) and DASP3 (right) MISST groups with known Prx isofunctional groups. The box color indicates the percent of the subgroup identified by each DASP/DASP2 or DASP3 group, where white indicates 0% of the subgroup identified and black indicates 100% of the subgroup identified.

**References**

1. Huff RG, Bayram E, Tan H, Knutson ST, Knaggs MH, Richon AB, et al. Chemical and structural diversity in cyclooxygenase protein active sites. Chem. Biodivers. 2005;2:1533–52.

2. Huff RG. DASP: Active Site Profiling for Identification of Functional Sites in Protein Sequences and Structures. 2005.

3. Nelson KJ, Knutson ST, Soito L, Klomsiri C, Poole LB, Fetrow JS. Analysis of the peroxiredoxin family: using active-site structure and sequence information for global classification and residue analysis. Proteins. 2011;79:947–64.

4. Bailey TL, Gribskov M. Combining evidence using p-values: application to sequence homology searches. Bioinformatics. 1998;14:48–54.

5. Akiva E, Brown S, Almonacid DE, Barber AE, Custer AF, Hicks MA, et al. The Structure–Function Linkage Database. Nucleic Acids Res. 2014;42:D521–30.
